# Transformation of Children’s Paintings into Public Art to Improve Public Spaces and Enhance People’s Happiness

**DOI:** 10.3390/ijerph192416780

**Published:** 2022-12-14

**Authors:** Na Luo, Rahinah Ibrahim, Sazrinee Zainal Abidin

**Affiliations:** 1 Faculty of Design and Architecture, Universiti Putra Malaysia, Serdang 43400, Malaysia; 2 School of Education Science, Baise University, Baise 533000, China

**Keywords:** public art, children’s paintings’ characteristics, public space, people’s happiness, sustainable design informatics

## Abstract

Characteristics of children’s paintings have been suggested considered for application in public art since they are known to positively evoke a sense of well-being when people see them. This study aims to understand the impact of artistic features from children’s drawings on people’s well-being; then analyzing the adaptive design principles of 3D public art featuring children’s paintings on people’s happiness; and finally, exploring the influence of 3D public art featuring children’s paintings on improving public spaces and enhancing people’s well-being. The results lead to proposing a conceptual framework for public artworks in public spaces for improving people’s happiness. The proposed conceptual framework recommends that, by applying the visual and thinking features of children’s paintings to public art, artists can design high-quality artworks suitable for a city, which could improve people’s happiness in public spaces. This study recommends further research into how public art can promote public spaces and shape the urban culture. It contributes to enhancing the quality of public art and public spaces, and inspiring a sense of well-being among citizens through the use of appropriate public art. The results are significant because they will help artists to create more high-quality public artworks for urban public spaces in order to evoke people’s happiness.

## 1. Introduction

Through the progress of times, public art increases its appearances in people’s lives and studies. Public art plays an important role in urban culture, urban spirit, and urban life. A good public art design can not only enrich our city’s appearance but also enter people’s hearts through art media. However, there is lack of commercial interest in, and authority guidelines on, how artists could display their artifacts in the public spaces. Without much support, artists will have problems in promoting contemporary Chinese public art [1].

Children’s paintings can arouse public happiness through visual means and bring infinite creative inspiration to artists. Modern art legends such as Paul Klee (1879–1940) and Pablo Picasso (1881–1973) had recognized the influence and inspiration of children on modern art as they broke through the intensely iconic phases of Primitivism, Abstraction, and Expressionism [2]. Almost all modernist artworks were criticized as being boring, empty, and unattractive to the general public [3]. Here, the authors are motivated to develop a process that translates the characteristics of 2D children’s paintings into 3D public art. This study believes it will help to enrich the artists’ creative thinking, make public art more approachable, and provide a greater sense of well-being by enhancing people’s interaction and engagement with public art. In the process of gradually improving the quality of life, citizens have higher requirements for the urban environment and public cultural space, which consequently promote the development of urban public art. Therefore, due to the current digital age, this study finds it necessary to study and explore the development and creative methods of urban public art. Hence, cities are recommended to consider children’s paintings for urban public art since they are known to positively arouse people’s pleasure when they see children’s paintings.

Studies into children’s paintings and public art have largely been based on their own independent research. Public art is art for the masses. The frank, childlike language of children’s paintings is a visual experience that could be missing from our adult spiritual lives. In response to this situation, the aim of this study is to apply the characteristics of children’s paintings to 3D public art, to enrich the artists’ creative ideas and methods, and to allow urban public spaces to be improved and inspire joy. The art of children’s painting are expected to help enrich the visual language of public art, evoke adults’ fondness towards their personal childhood memories, and even find moral support for the relief of stress when they are under tremendous pressures. This paper explores whether public art with the characteristics of children’s drawings can increase the engagement and interaction between people and public art, thereby inspiring a sense of well-being.

## 2. Research Methodology

The literature review in this paper follows the “systematic literature review synthesis process” [4]. This process is an independent type of literature review [5,6,7] to determine the background theoretical context through an understanding of selected existing literature in the early research conceptualization phase. This study used Ibrahim’s research question (RQ) construct classification technique to identify three different RQ constructs, namely the “WHO”, “WHAT”, and “HOW”, in formulating the primary research questions [8].

The “WHO” is defined as the element impacted by the research, the “WHAT” refers to the body of information or knowledge needed to solve the problem, and the “HOW” is the targeted impact of the study [9]. This study sets the characteristics of children’s drawings as the “WHAT”, the transformation into 3D public artwork as the “HOW1”, and the identification of public spaces as the “HOW2”. This study had chosen to explore the ‘WHAT’ and ‘HOW’ constructs. This study seeks to answer the following research questions: (1) What are the influencing factors of 2D children’s drawing features on people’s well-being?; (2) What are the design principles of 3D public art with 2D children’s paintings’ characteristics that can improve people’s happiness?; and (3) What is the impact of public art with 2D children’s paintings’ characteristics on urban public spaces and the urban cultural atmosphere? Based on the research questions and research objectives of this study, a bibliographic search was conducted in WOS, SCOPUS, and Google Scholar databases with these keywords: public space, public art, children’s paintings’ characteristics, and personal subjective well-being.

**Figure 1:**
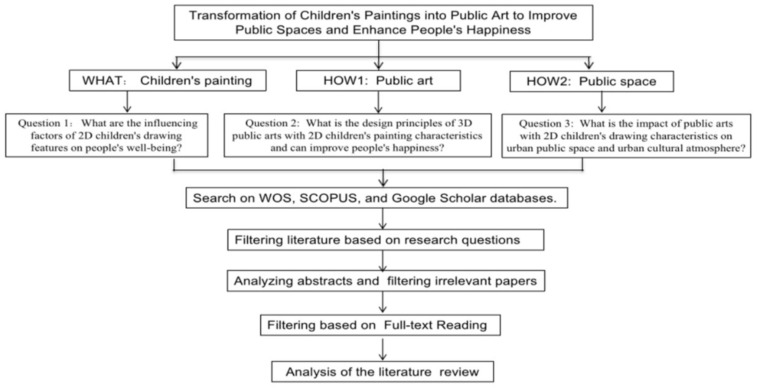
The workflow of the systematic literature review synthesis process adapted with permission [9]. Copyright 2018, Ibrahim and Mustafa Kamal.

The abstracts of the selected journals were later reviewed in terms of their main content and conclusions, how their works would support future research, and what areas would need strengthening. The 87 abstracts were selected for detailed review before being assigned to specific sub-themes according to the importance of their existence. The results of this exercise then generated a synthesis summary for each major theme, which was further cross-analyzed to integrate possibilities and prioritize synthesis summaries to analyze the impact of public artworks featuring children’s drawings on public space and people’s well-being. The key synthesis extracts were formed using the EAGLE Navigator online system by following the steps in the Point of Departure (POD) tree diagram as well as the documentation of the synthesis process. Figure 1 shows the paper’s flowchart of the literature review methodology.

## 3. Results and Discussion

### 3.1. What Are the Influencing Factors of 2D Children’s Drawing Features on People’s Well-Being?

Children’s drawings are works produced by children at an early age, after observing things in the real world, through their own imagination and free play, using drawing, doodling, and other means. The study of children’s drawings dates back to the late 19th century [10]. Children’s drawings are similar to a form of play that grows with them. Children’s drawings reflect their innocent and pure nature and are a special way for children to express their inner feelings and thoughts [11]. This study will first analyze the thinking and visual characteristics of children’s drawings and later will explore the impact of the visual characteristics of children’s drawings on people’s sense of well-being.

#### 3.1.1. Thinking Features of Children’s Drawings

Drawing is seen as a symbol that children use to communicate and is equally important as a visual language. Drawing is a communicator of self, while drawing is a processor of knowledge and a process of play [12]. In essence, children’s drawings reflect their upbringing and psychological condition. The characteristics of these drawings change as the child grows, and children translate their perceptions into images that people can understand and observe [13]. Research on how young children’s drawings change and develop is well documented, with a large body of literature on the field dating back to the 19th century. However, most of this literature focused on the developmental aspects [14].

There are typical visual features in children’s drawings [15]. Numerous studies have shown that children learn about their environment and nature from direct experience. Children show a high degree of randomness in the drawing process. Children’s drawings involve their imaginative, expressive, and creative abilities [16], and during the drawing process, the children’s perspectives are fluid. Children’s drawings also do not have a fixed shape; they add their favorite images at will. Hence, the development of their drawings is related to their cognitive abilities [17].

Since the 1990s, academic research on children’s art has become more advanced, in their studies, scholars analyzed in detail the features of children’s thinking and the exaggerated expressiveness of their drawings [18]. Children’s drawings contain the primary features of the depicted objects, which indicates that children can observe [19]. In other words, this means that the child would retain the most basic structural features of the object in the drawing process. All shapes represent a symbolic character. However, scholars’ understanding of children’s drawings varies considerably, depending on the different cultural backgrounds of the scholars [20].

The random and fun nature of children’s drawings can stimulate children’s enthusiasm and interest in creativity and can help children improve their imagination, creativity, and observation in the process of drawing [21]. Drawing is imbued with personal creativity by children where drawing is one way to express people’s imagination [22]. Children’s drawings are influenced by the drawing environment and drawing materials, and children’s drawings are derived from their life and drawing experiences [23]. The distinctive characteristics of children’s drawings can be observed during the silent, symbolic, and creative periods. 

Although scholars and artists have studied children’s drawings in depth, the current findings suggest that there is considerable controversy in scholars’ understanding of children’s drawings and children’s art. Carothers argues that, although children’s drawings have been studied from several perspectives by previous authors, their status as works of art has not been considered [24]. Metin offers his own unique perspective, arguing that gifted children and normally developing children have similar drawing characteristics while acknowledging that the differences between boys and girls are not significant and that the differences between children’s drawings are age-related [25]. Barraza shares Mine’s view that children with significant cultural differences show more similarities than differences in their drawings [26]. However, Metin only selected a small number of children for his study and did not mention more regions and countries [27]. Similarly, Barraza only focused on children’s drawings in the United Kingdom and Mexico [28], and the study would have been more comprehensive if it had covered more regions.

In general, this study agrees with Hodgson and Khorshidi that it is important to respect and understand the characteristics of children’s thinking in drawings, such as exaggeration, randomness, and creativity, and to analyze the characteristics of children’s drawings [13,15]. Children break through stereotypes and are naturally positive. They also express their emotions and personalities through art. Moreover, Kudryavtsev’s point of view is supported [21]. The randomness and fun nature of children’s drawings are the driving force of children’s creativity. Thus, based on Wright’s view, this study will focus on the types of children’s drawings, artistic expressions, and artistic characteristics [27]. Children’s ability to “borrow” sensory and emotion-based language to metaphorically describe ideas is common, not only in the visual arts, but also in drama, dance, and music [27]. Finally, this study explores the randomness, exaggeration, symbolism, uniqueness, and playfulness of children’s drawings to increase our knowledge of the artistic thinking process.

#### 3.1.2. Visual Features of Children’s Drawings

The process of children’s psychological development influences the content in children’s drawings, and the drawings of children of different ages reflect their understanding of visual information [28]. The content of a drawing determines its quality, which is represented by the composition, line, color, and shape [29]. Hence, children’s drawings have their own characteristics in terms of expression.

The development of children’s painting is closely related to the psychological development of children. Simplicity and generality are the more prominent features of children’s paintings, and the linear development of children’s paintings has obvious differences in different age groups. Differences in children’s paintings are closely related to children’s long-term living environment and personality characteristics [30]. Children’s drawings have their own unique characteristics of shape, color, and composition. The simplicity of generalized shapes and bright colors as well as the free and random composition in children’s paintings are appealing [31]. When young children first begin to draw, they are less expressive [32]; as a result, their drawings are simple in shape and lack details, moreover, the objects in their drawings are mostly composed of lines. There will be obvious differences in the graphics and tools that children use when drawing the same object, and their artistic characteristics can be classified and studied during the research process [33]. 

Semiotic art in children’s drawing can realize communication without borders, and semiotics can help analyze the art of children’s drawing in art education [34]. Typically, 2- to 5-year old children draw people like tadpoles. Children between the ages of five and seven draw people like sticks. By the time a child is 7 to 9 years old, the figure is more complete, and the characteristics of the figure’s gender and costume can be distinguished [12]. Color is the first impression of the world that people perceive visually, and children are no exception. When children observe an object, they first analyze the color characteristics and, then, have a specific understanding of the whole thing through the color characteristics [35]. Children always pay special attention to the color of things, and bright colors attract children’s attention [36]. Children’s paintings boldly use contrasting colors that are composed of high-purity colors without mixing. Bright colors and high-purity colors are in line with children’s aesthetic feelings [37].

The main characteristics of messy compositions are having no fixed arrangement in the pictures and people can draw according to their own feelings. Children have poor control over the size of objects and ignore the details [38]. Children often use lines to divide their paintings, which forms a unique way of composition. The main feature of the baseline composition is to draw a baseline under the drawn shape, such as a line to separate the ground from the sky. This means of composition makes the picture to start becoming orderly. The appearance of a baseline composition indicates that the child has the idea of arranging elements in a sequence. As the children develop, thematic compositions would emerge in the painting. By arranging the main image and the secondary image, the main expressed content is highlighted, thus forming the theme of the picture and expressing the artist’s own ideas. Lijima, however, differs slightly from other scholars in his view that there are gender differences in children’s freehand drawings in terms of subject matter, color, character composition, and expression [39]. Boys prefer to paint moving and mechanical objects in dark or cool colors and often use bird’s-eye compositions when painting, while girls prefer to paint figure patterns (especially girls and women), flowers, and butterflies, use light and warm colors, and tend to arrange patterns in succession on the ground [39].

There are some questionable assumptions in the study of children’s artistic development. One view is that children’s drawings follow universal patterns in their early years, regardless of their culture or gender, and that the patterns of children’s artistic development do not change in the early stages of so-called primitive art, regardless of where they were born. Piaget analyzed the universality of children’s artistic development based on cognitive development, and that the universal patterns present in children’s drawings in all cultures and countries vary between stages change [40]. In terms of the visual characteristics of children’s drawings, this issue is still not fully explored, despite several studies having been conducted by researchers. At the beginning of the 20th century, studies on children’s drawings were focused on visual data: space; line; perspective ability; etc. [27,37,41]. In terms of the visual characteristics of children’s drawings, Soylu only focused on describing the patterns and characteristics of children’s drawing line development, without discussing other elements of their drawings [30]. In contrast, Turkcana focused only on using semiotics to analyze elementary school students’ drawings, which would have been more scholarly if the study had been broader in scope [34].

In general, this study agrees with Rubenstein in summarizing and analyzing the modeling features (such as simplicity, generality, flat modeling, parallel vision, independent modeling, and positive perspective expression), color features (such as bright colors, bold contrast, high purity), and composition features (such as messy, baseline, thematic compositions, free, casual, and highlighting the theme) of children’s paintings [31]. This study proposes to follow Soylu in examining children’s paintings and analyzing the elements and characteristics of children’s paintings [30]. Additionally, this study proposes to explore the visual characteristics of children’s painting art such as simple and independent shape, bold contrast, and high purity of color, and free, casual, and prominent subject composition, which are conducive to the wider application of many elements of children’s painting in art creation.

#### 3.1.3. The Impact of Children’s Drawings on People’s Subjective Well-Being (SWB)

SWB is defined as well-being in terms of pleasure attainment and pain avoidance, and it is closely related to hedonic mental states as it depends on how an individual feels at certain specific times [42]. At the beginning of the twentieth century, children’s art became the subject of serious study by scholars, enabling it to be considered as art [43]. Art has a unique charm, and works of art not only enrich and regulate people’s spiritual life, but also relieve various social pressures [44]. As an important field of art, children’s painting is inextricably linked with people’s happiness.

Happiness is one of the oldest themes in philosophical research [45]. The various definitions of the overall happiness of individuals have been understood differently at separate times. The question of how to define one’s overall happiness refers back to the philosophical debates of ancient Greece; “happiness” versus “hedonism”. The school of hedonism, which was led by Epicurus, believed that happiness came from the enjoyment and pleasure of life [46]. Children’s drawings are known to induce feelings of relaxation and pleasure. Therefore, visual art, such as children’s drawings, can stimulate a sense of well-being [47]. The creation of children’s art is not an individual endeavor and questions the assumption that artworks are mirrors of children’s thoughts [36]. Art is often thought to enhance people’s well-being [37]. There is an interplay between children’s art and avant-garde art. The participatory nature of children’s art is particularly emphasized by the artist Paul Klee, who collected works created by children and saw them as a path to art [48].

The surrealist elements in the paintings of the famous artist Miro are inextricably linked to children’s art, thus opening up new territories. Most of the artist’s works are characterized by simple shapes, a child-like freedom of expression, and a high degree of spontaneity and randomness, through the use of things stored in his memory since childhood [49]. Children’s paintings have attracted the attention of artists with their honest childishness, vivid colors, and unbridled imagination. Children’s drawings not only occupy a unique place in the field of art, but they can also be visually enjoyable [18].

The innocence and naturalness of children’s paintings give people positive emotions, so children’s paintings can influence people’s happiness from the visual aspect [50]. Children’s paintings are the most primitive and valuable way of presenting the mind. If primitive art is the origin of all art, then children’s art can be said to be the true embodiment of the artist’s heart. There is no doubt that innocence and childishness are very positive. They naturally resonate with people, bring them into a state of deep relaxation and freedom, and also bring them a sense of well-being. Innocence and childlike interest are the source and goal of artistic creation [31]. There are many similarities between the works of modern art masters and children’s paintings [27,33].

Huang Shanwu, a famous Chinese public artist, said in an interview with the first author, “There is no doubt that children’s paintings can bring joy to people. I aspire to think like a child without any complicated emotions when I create. I want to express what I want to express in a simple and pure way. I like to work in an uninhibited way, somewhat like children’s painting. In my own work, for example, I look for particularly bright lines and light, and I often use very simple shapes to convey the charm of the art”. However, the art of children’s painting can not only enhance the happiness of adults, but also help children to form a unique aesthetic concept and improve their aesthetic ability, as well as satisfy their need for self-expression of emotions, which is also important for enhancing children’s happiness [51]. 

The current research results show that there is a great deal of controversy about the actual meaning of children’s paintings. However, not all scholars and the general public believe that children’s drawings are valuable. Some people think that children’s drawings are difficult to understand, and there are no figurative images and high-quality lines in children’s drawings. Children’s drawings are messy in composition and do not clearly express the subject matter of the drawing. Painting is a way for children to communicate with the world. The unique form of expression may go beyond the emotions that language can express and empathize with the public. Nowadays, under conditions of fast-paced lives and pressure, people prefer to see simple, pure, and connotative art forms that can create emotional resonance and enhance their sense of well-being [52].

However, in considering the differences between children’s paintings, the public recognizes children’s art and celebrates adult art. This study believes that it is possible to be happy with children’s artworks, because in their artworks we can see the very first signs of future achievements. The artistic value of children’s work is determined by the viewer’s perception such that educators see it as a way to see through aesthetic development, psychologists see it as a key to understanding children’s behavior, and artists see it as the most direct expression of children’s inner emotions. However, educators, psychologists, and artists alike are recommended to see children’s artistic development as a unique and integral part of their overall education [53]. According to Gardner, “it is the child’s approach to art, the child’s subconscious sense of form, the willingness to explore and resolve emergence, the ability to take risks and the emotional needs that must emerge in the symbolic realm” [53].

In general, this study supports Węziak Białowolska that people’s subjective well-being comes from enjoyment and pleasure in life [37]. This study recommends Totterdell’s view that children’s drawings not only occupy a unique place in the field of art but can also be visually pleasing [47]. This study also promotes Khayif and Kümmerling’s view that children’s drawings can provide creative ideas and inspiration for artists. This study agrees with Wang Likun’s view that the art of children’s paintings can provide a new source of inspiration for artworks [52]. By analyzing the artistic characteristics of children’s paintings from four perspectives: point, line, surface, and color, this study concludes that its strong generalization, imaginative creativity, and combination with elemental modeling, color use, composition, and expression in other art forms can be realized if based on the artistic expression of children’s painting. This study posits that children’s paintings not only provide an artistic experience but can also relieve people’s mental stress, inspire artists, and stimulate a sense of subjective well-being through visual images.

#### 3.1.4. The Process of Conceptual Framework Formation

**Figure 2:**
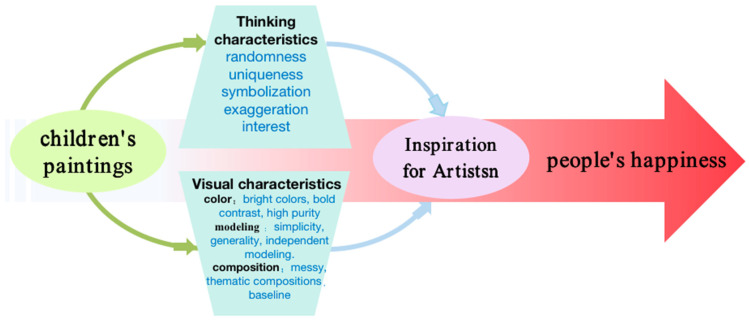
Proposed conceptual framework for influence of children’s paintings’ features on people’s well-being.

This study aims to explore how children’s paintings can arouse people’s happiness. This paper records the analysis of the thinking and visual characteristics behind children’s paintings to determine which type of children’s painting is likely to arouse people’s happiness. The results show that exploring the thinking characteristics of children’s paintings, such as randomness, exaggeration, symbolism, uniqueness, and interest, can increase the cognition of children’s painting art thinking-process. The study proposes to explore the visual characteristics of children’s painting art (such as simple and independent modeling, bold color contrast, high purity, free, casual, and prominent theme composition) and to apply them to wider applications in art creation. As an important category of art, children’s paintings can provide artistic experience to relieve people’s mental pressure, stimulate artists’ inspiration, and stimulate people’s subjective well-being through visual images. The results show that the characteristics of children’s paintings can stimulate people’s happiness and have a profound impact on art. Adding the characteristics of children’s paintings to the artist’s artistic work can increase the artist’s creative materials and also improve the creativity of the artist’s works, thus stimulating the joy of the audience. In summing up, this study proposes to focus on analyzing the specific characteristics of children’s paintings in two areas—thinking and visual characteristics—that would inspire artists hence arouse the public’s pleasurable happiness through visual means. Figure 2 is the proposed conceptual framework.

### 3.2. What Are the Design Principles of 3D Public Arts with 2D Children’s Painting Characteristics and Can They Improve People’s Happiness?

Children’s paintings not only occupy a unique position in the field of art, but they can also be visually enjoyable. The characteristics of children’s paintings can enhance people’s subjective sense of well-being. This study questions whether public art with the characteristics of children’s drawings will inspire subjective well-being? In this section, the study first examines the best design principles for public art that transforms children’s drawings into 3D public art. Then, it examines the characteristics of children’s drawing elements that influence public art.

#### 3.2.1. The Best Design Principles for 3D Public Arts

Although contemporary public art emerged in Mexico and the United States in a self-conscious state at the beginning of the 20th century, it did not enter the phase of theoretical research then [54]. It was not until the 1980s that the concepts of the public sphere and advocacy were widely and intensively discussed. Exploration of the evolution of public art started in 1989 with the emergence of contemporary public art history. In 1987, the establishment of the Public Art Review marked the entry of public art into the level of theoretical research. It was the same year that the Tilted Arc of the American artist, Serra, was moved out of Federal Plaza in lower Manhattan in response to the public outcry, which consequently became a famous event in the history of public art [55]. An open space, that has the characteristics of openness and allows for free public participation and recognition, is known as a public space and should not have an imposing barrier within it.

With the development of the times, public art is no longer limited to murals, urban sculpture, and architecture in urban spaces. It now includes festivals, exhibitions, events, or the spirit of urban culture. The impact of public art on the visual characteristics and emotional evaluation of the landscape argues that different public art would produce different evocative visual characteristics. This study posits that visual characteristics are often associated with pleasure. Therefore, public art helps to improve urban life and the image of the city [56]. The creation of public art must be in keeping with people’s aesthetic sensibilities and be artistic. Public arts are based on aesthetic forms and convey the concept of beauty and emotions to people through their images, textures, textures, colors, and other compositional elements, thus infecting the public and making them enlightened and feel beautiful [57]. Public art should not only meet basic aesthetic requirements in terms of form but also meet the daily needs of the public in terms of function [58].

This refers to the artistry and functionality of modern urban public art. As an important part of the urban landscape, modern urban public art is the link between people and the urban environment. Therefore, the urban public art design is both artistic and functional [1]. Modern urban public art should have multiple functions, such as practical, aesthetic, and symbolic. In environmental art design, it is more important to address the functional design and design functions of public art in urban environments. Artists are recommended to follow design principles when designing high-quality public artworks [59].

**Figure 3:**
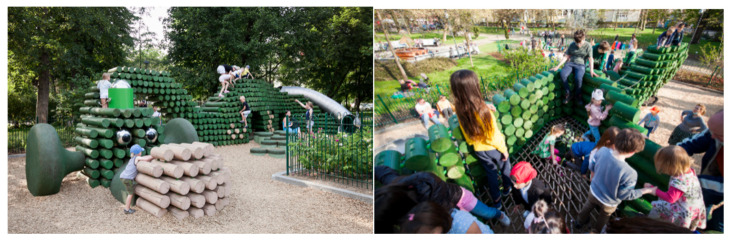
Lza Rutkowska Pri’s work “Dragon.” Reprinted with permission from Ref. [62]. Copyright 2021, Lza Rutkowska Pri.

The word “public” in public art indicates its propaganda nature. The meaning of public refers to public spaces and their relationship between people, space, and society. Therefore, design must consider the public perspective. The philosopher Hannah Arendt (1906–1975) pointed out that ‘publicity’ is not a random thing placed in a public space; it is something that is seen and heard by everyone in a public place, such as a table is seen by many people. Moreover, public art has a broader human dimension, and its continuity depends on its being appreciated and accepted by people from all over the world, from different cultural backgrounds, and from different generations. The daily aesthetic phenomenon of the public and its influence on public art could start with the current phenomenon of urban public art design. Inspiration is sought from public issues to make public art truly integrated into the public environment, to inject the aesthetic communication value of public art design, to improve the aesthetic level of public life, and to promote the development of urban culture [60]. The relationship between public art and its audience is one of sharing and communication, which reflects the most important characteristic of public art advocacy. The rationalization of social organization and the embodiment of civil rights are essential to achieving advocacy. Propaganda can be the core content of public art and also become the basis for its creation. Public art punctuates the urban form and is an important part of the urban landscape [61]. An example of this is the work of Polish artist, Iza Rutkowska Pri, with the “Dragon,” which was built in the park of Maria and Lech Kaczyński (see Figure 3).

For a long time, art has dealt with the public from an overlooked perspective. The current research finds that interaction technology is becoming more mature, and the forms of interaction in the urban public environment are also increasing. The unique choice needs to be integrated with the spatial environment and regional culture, which is also the basis for the rational existence of the theory of public art interaction. Participation is one of the most important characteristics of public art. The behavior of the audience facilitates the aesthetic value of public art and makes urban public art a vital component of contemporary public art. In the specific development of modern urban public art, forms of external interaction include physical interaction, emotional interaction, and conceptual interaction [63]. With the continuous high-quality development of modern information interaction technology, the presentation of public artworks has gradually shifted from a static to dynamic presentation, from an indoor to outdoor presentation, and the level of transformation of the modern urban environment in the specific interaction process has also shown a trend of continuous improvement [64].

In public art, art is the essence, thus, the first thing it embodies is its artistry and innovation. Public art interventions attempt to produce inclusiveness. Public art is seen as an aspect of cultural rules, which has sparked resistance [64]. It goes without saying that a work of art can only be considered a work of art if it has a certain degree of artistry, and the vitality of a work of art lies in its constant spirit of innovation. The principle of creativity in art is an important principle that must be followed in design activities [65]. Public art design is no exception, so it is necessary to introduce the principle of artistic innovation into public art design [66]. The design of public art is recommended as a conceptual plan. A good, distinctive idea, with an intelligent entry point, is often the key to its success. Therefore, it requires designers to break through, innovate, change old concepts and forms, and open up new spaces for development.

**Figure 4:**
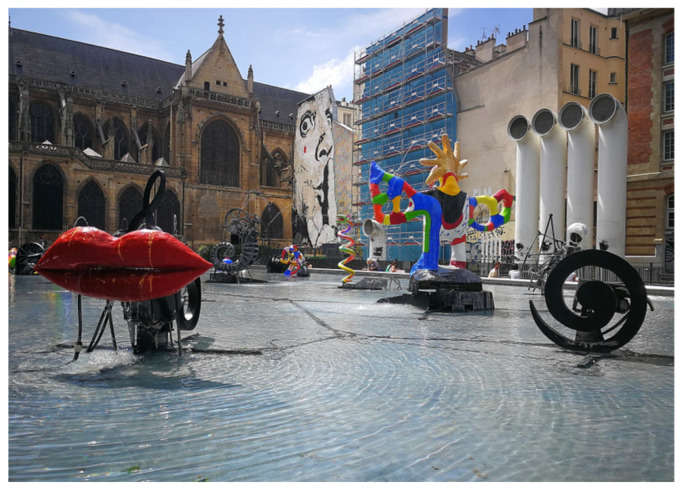
LaFontaine Stravinsky reprinted with permission from [67]. Copyright 1983. Niki de saint Phalle and Jean Tinguely.

Public art design should be seen as part of urban planning and design, and it is inevitably closely linked to nature, the city, architecture, and gardens, and it belongs to and is integrated into the city. Public art with the characteristics of children’s paintings should be integrated with the spatial environment. As part of the urban environment, public art is usually a subordinated to the overall urban style. It would be a cultural feature of the city that completes and enriches the whole. This study emphasizes that public art is not a crude interference with nature or an imposition on the environment, and should be integrated into the spatial environment. Public arts in urban squares combined with the environment need to be considered in harmony with the architecture, and to echo it. For example, in Figure 4, the LaFontaine Stravinsky fountain is the work of the famous French artists Niki de Saint Phalle (1930–2002) and Jean Tinguely (1925–1991) [67]. They used this fountain to express the musical style of the composer Stravinsky. The unique artistic style is well-integrated and echoes the architecture of the adjacent Pompidou Centre for Art and Culture. The urban public artworks not only reflect their own charm, but also the beauty of their context, which brings a new experience to life.

The safety of the work is one of the most fundamental principles of public art and landscape architecture design [68]. It includes consideration of the structure’s load-bearing capacity, material performance and quality, waterproofing, slip resistance, fire protection, protection against electric shock, and protection against light pollution. It even includes consideration for potential hazards that may exist, such as whether public artworks installed in urban street areas may affect public transport and pedestrian traffic, and whether road corners may block the view of moving vehicles and cause traffic accidents. Different types of structures and public art designs have different safety requirements, so not only is a high level of awareness of safety design required before design, but relevant design standards and codes need to be carefully reviewed and used as an important basis for design [56].

The current urban public art construction has ushered in a new period of development. The public’s perceived attractiveness of public art venues varies from place to place. For a city, without public art, it would be boring. Public art has a significant role in urban space, and different public art in cities has distinct roles and artistry [69]. Public art is the embodiment of modern urban culture, the ideals and passions of urban life, a unique way to shape, remember, and enjoy the city. It is a symbol of the geographic location and regional identity of the city’s cultural spirit [70].

However, most of the above-mentioned studies are independent studies and there is dearth of comprehensive studies. Examples include the benefits of environmental art and the role of art in the environment [71]; the impact of public art on the visual character and emotional evaluation of urban landscapes [55]; and a critical discussion of public art created by artists around the world in urban public spaces [69]. Public art created in urban public spaces has received attention and critical discussion from artists around the world. Scholars have focused on different areas, ranging from scholars who delve into public artworks in a neighborhood [72] to scholars who focus on the function of permanent sculpture in urban spaces in relation to the urban landscape and art education. Another is on public sculpture making urban spaces more urban and making art more accessible to the public [73]. Some scholars have also focused on the impact of urban green spaces and urban spaces on people’s well-being [74].

In general, this study agrees with Russell that public art, by its very nature and source of creation, has from the very beginning exuded a strong desire to satisfy public needs [55]. Contemporary public art is mainly a reflection of the public spirit and a reflection on public affairs, and “publicness” and “participation” are two of the most important characteristics of public art. This study also recommends Yan’s view that public art is an important language for modern urban design, and that urban design is the basis for public art, as well as a variety of forms of art [57]. Finally, the public and interactive nature of public art are the basis for the creation of public art and being the core content of public art. Public art carries the culture of a city and can enhance its image. The creation of reasonably designed and aesthetically pleasing public art could be guided by publicity, interactivity, artistry, functionality, integration with the environment, livability, and safety. Excellent public artworks are recommended to be attractive and contain regional cultural elements that could provoke a sense of belonging and well-being.

#### 3.2.2. The Relationship between the Characteristics of Children’s Painting Elements and Public Art

Public art is a creative theme mobilized to form the image of a city. Public art can be understood in two important ways. First, art has become a symbol of progress and development, and secondly, art has become a conduit for criticism and protest against these forces [75]. Many artists believe that this simple and direct expression in children’s drawings is closer to the original state of artistic expression. This pure approach has given the children new strength and inspiration. The legends of modern art, Paul Klee (1879–1940) and Pablo Picasso (1881–1973) recognized the influence and inspiration of children in modern art when they broke through the intensely symbolic stages of primitivism, abstraction, and expressionism. For Klee and Picasso, there was striking visual evidence that children’s pictorial elements were very appealing [2]. Some of their paintings do have a childlike style.

Further research revealed that the technique of incorporating interesting elements of children’s art was not unique to Klee nor Picasso; many prominent artists of the early 20th century also found a connection to primitive cultures and the appeal of children’s art. The artist’s borrowing and imitating the expressive forms and the painting techniques of children’s paintings was only part of the picture; what the artist most desired was to find a simple, sincere, and pure form of art from children’s painting and return himself to his most primitive state. The characteristics of Paul Klee’s artworks and the relationship between his works and children’s paintings are obvious, as Paul Klee’s paintings are full of childlike interests.

The key to the creation of public art is to show the characteristics of the artists’ and designers’ artistic styles and to avoid the phenomenon of a “one-size-fits-all” landscape. Originality means that the work is original, not copied or imitated, and has a unique personality in terms of content and form. However, originality does not mean advocating unjustified whimsy and not all scholars hold the same view. Some scholars believe that originality is not opposed to the reproducibility of works. Rather, it expands the influence of artists and designers and increases the value of the works themselves through legitimate copying and gifting.

Studies have shown that the creativity of children’s paintings is different from creativity in the general sense. From the nineteenth century, artists began to consciously study and promote children’s paintings and child psychology, and let many elements of children’s paintings be widely used in art creation, and children’s paintings gradually began to play an important role in the field of art. The creation of children’s paintings is called the “primordial drive of life” and is the manifestation of human instinct. Compared with adults, children’s thinking is more simple and more innocent, so drawing on children’s paintings and experiencing children’s thinking is a true experience of creation. Children do not have the means to acquire a systematic professional knowledge of painting, but the works they create through their brushstrokes are often unexpected and moving.

The art of children’s paintings refers to the artistic style characteristics of the category of children’s painting, as explained by Bian Xia in *Children’s Art and Art Education.* The discovery and understanding of children’s innocence and frankness can make people feel that they have returned to simplicity and found the value of simplicity [3]. Children construct forms directly from the depths of their emotions, and they are more creative than their imitators. At the same time, the simple and innocent aesthetic elements of children’s paintings can be fully exploited and applied to the creation of public art design, thus expanding new creative methods and expressions for public art design. Herewith, this study finds a practical significance in the children’s approach.

**Figure 5:**
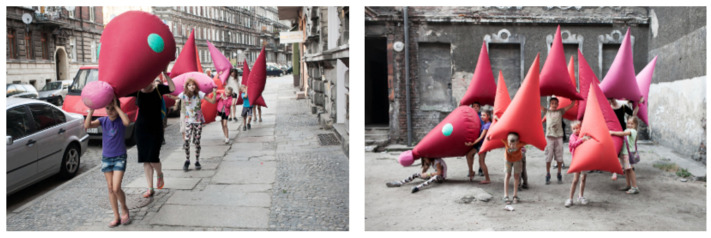
Lza Rutkowska Pri’s work “Hedgehog” in “Everyone’s Backyard” project. Reprinted with permission from [77]. Copyright 2015, Lza Rutkowska Pri.

However, not all scholars agree that children’s paintings can have a positive impact on public art. The messy compositions and overly complex colors of some children’s paintings are not suitable for use in public art. So, one should be selective in presenting artwork. Excellent art should be innovative. Creating new images is an important aspect of the public art design, so public art should be innovative. If a work of public art does not stimulate any imagination nor any evocation in the audience, it can only be an empty work without any artistic charm. The application of creative thinking in public art design can capture the viewer’s attention and make it resonate emotionally [76]. For example, in the project “Everyone’s Backyard”, the artist Lza Rutkowska Pri designed an 8-m-long “Hedgehog” (see Figure 5) consisting of individual spikes, whose simple shape and bright colors were loved by everyone.

Color has great significance for human beings. It is at the heart of visual aesthetics and has a profound impact on our emotions. The bright and vibrant colors of children’s paintings can make public artworks visually stimulating to the viewer. They resonate with the viewer. Applying the exaggerated proportions and exaggerated shapes of children’s paintings to public art can quickly attract the public’s attention, enhance the artistic effect of the work, and highlight the ideas of the creator. The use of symbolism is the application of this simple and general expression in children’s paintings to public art to convey to the audience. These are excellent public works of art with the character of children’s drawings. They are not only popular but also interact well with the public and inspire a sense of well-being.

The characteristics of children’s drawings give artists unlimited inspiration and creativity [78]. Children’s paintings are an artistic creation based on the nature of children. They contain innocence and purity, which can awaken a sense of purity and resonate with people. Through this liberation of the mind, children’s paintings are an artistic value in themselves, and they possess great inspiration and connotation that can stimulate people’s imagination and create a good creative environment [79]. Finally, incorporating the characteristics of children’s paintings into the creation of public artworks can enhance the artistry and creativity of public artworks, as children’s paintings can provide new strength and inspiration for artists.

#### 3.2.3. The Process of Conceptual Framework Formation

**Figure 6:**
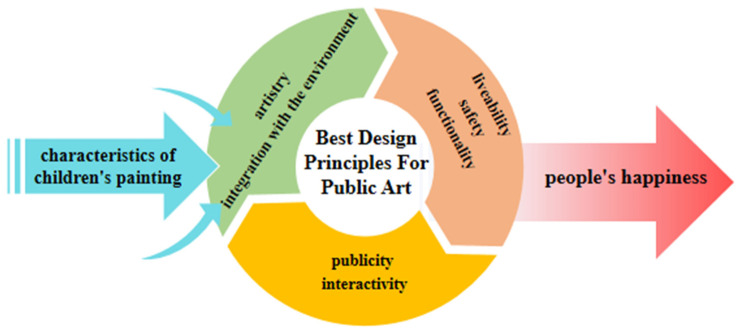
Proposed conceptual framework for design principles of public artworks.

The reinterpretation of children’s painting elements with public art in public spaces can create a positive and inspiring environment because they resonate well with people. For this reason, this study has no doubt that children’s paintings occupy a unique place in the art world and can also be visually pleasing. The purpose of this section is to explore the best design principles for transforming children’s paintings into 3D public artworks. This study found that well-designed and aesthetically pleasing public art creations are recommended to follow the following principles: publicity; interactivity; artistry; functionality; integration with the environment; livability; safety. Additionally, excellent public artworks should have attractive regional cultural elements that inspire a sense of well-being. In the second part of the study, it was found that incorporating the characteristics of children’s paintings into the creation of public artworks can enhance the artistry and creativity of public artworks, as children’s paintings can provide new strength and inspiration to artists. However, the promotion of and interaction with public art is not only the foundation of public art creation, but also the core content of public art. When people see excellent public artworks with strong innovation and artistry, they can inspire a sense of well-being within themselves. To summarize, the creation of excellent public artworks can enhance people’s happiness by following the principles of publicity, interaction, innovation, integration with the environment, livability, and safety. The idea proposes that characteristics of children’s paintings could be applied to enhance the innovation and attractiveness of public art. Figure 6 is the conceptual framework for how design principles of public artworks could influence the people’s happiness.

### 3.3. The Impact of Public Art with Elements of Children’s Drawings on Public Spaces and People’s Well-Being

The influence of environmental factors and personal characteristics on subjective well-being has recently attracted considerable interest from academics and practitioners [80]. In this section, this study explores the impact of public art with elements of children’s paintings on public spaces and people’s subjective well-being. To achieve this research objective, the role of public space is first analyzed. In the next section, this study explores whether public artworks with children’s paintings in public spaces can stimulate people’s sense of well-being.

#### 3.3.1. Public Art and Public Space

Through the passage of time, worldwide exchange and communication have become easier, and so has the integration between cultures. Infusion of the ethnic influences has provided more content for the diversity of public art, and the development of public art design has been showing an increasingly diverse development trend. The functions of public art will also show diversity, such as monumental, decorative, practical, recreational, leisure, embellishment, and forecourt activities [80]. At the same time, public art, as an important part of the urban landscape, also aims to improve the urban environment, enhance the overall image of the city, enrich people’s spiritual life, and improve the quality of life. Public art is an extension and concretization of urban design and an important language and element of urban culture. Scholars have explored the limits of public space’s potential to enhance people’s well-being from different perspectives [81]. The most important characteristic of public art as environmental art is its “publicness”. First, urban public artworks are the product of modern society and a specific stage of historical development. Second, public artworks serve the public. Third, public art is set in the public open spaces of cities, and fourth, compared to other arts that may exist in private, enclosed spaces, they exist in an ever-changing historical cognitive process with respect to the form of time. Fifth, public art contains a sense of public dialogue, rational interaction, and the construction of a common spirit would give meaning and connotation of common spiritual construction [66].

In the macrocosm of public art and space, the link between them is a necessity. Public space acts on public art, and public art in turn acts on public space, a form of art that results from the interaction between public art and space. The urban landscape in the urban space is conducive to the presentation of public art [68]. Urban environmental design research emphasizes the quality of public space and its positive effect on people’s well-being [82]. Public spaces take many forms, from informal street corners to large municipal facilities. Public spaces have long played a vital role as the center of various settlements and the focal point of public life, activities, and events [83]. Urban public space is the main place of leisure and recreation for urbanites and it has an all-encompassing connection to the daily lives of urban residents [84]. The infrastructure of urban public space is representative of the city’s image because it reflects the development process of the city [85]. Moreover, the urban public space also occupies a critical position to improve people’s well-being [86]. The enhancement and improvement of urban public space help to optimize the quality of the urban environment and provide a pleasant experience for the residents [87]. Current research has already outlined the importance of the contribution of public space in the achievement of “human health and happiness”, and “ an attractive and livable city” [88].

In the process of urban construction and transformation, there are many problems in the public environment. Sustainable and inclusive public spaces need to be designed [89]. In the field of public space research, the author of [90] studied the relationship between public culture and urban public space; in [91], the author presented a detailed assessment of public space; and in [92], the author explored the relationship between art space, public space, and community development.

Excellent public artworks enrich public spaces while enhancing the image of the city and bringing new enjoyment to the viewer. Urban landscape aesthetics are a key component of overall urban design. However, although urban image design is by far the most traditional field of planning, it seriously lacks a cohesive theoretical foundation [93]. Public art and the image of urban public spaces together add tremendous value to the human experience and the development potential of cities [94]. Empirical studies tend to show that taking full advantage of public art in shaping the image of cities is important for promoting urban culture and urban development. Although public art is universal, open, and artistic, this study agrees that it has a distinctly different socio-cultural mission and value, and has become an integral part of urban culture; principles regarding the definition of good public-space design are often based on the intuitive analysis of artists [95].

Urban public art plays an extremely important function in shaping the image of a city [83]. It is an important vehicle and medium of transmission for urban culture. The design of urban public facilities should be guided by urban culture to ensure the harmony and unity of urban public facilities and urban culture, thus forming a unique urban image [86]. Professionals in different fields have studied urban image and public art to varying degrees. For instance, in a study about changes in public space, the author of [96] focused on the recent redevelopment of urban squares. However, some Chinese scholars have concentrated on the public art design-style of cities and the effect of public art in shaping urban culture. These scholars have something in common in that they had emphasized theoretical research alone.

Overall, this study is consistent with the position taken by Latham [85]. The increasing influence and role of public art on the image of the city require better use of the value of public art to connect public space and public life. This study recommends Rung’s idea that people and public art can communicate directly and up close. In art activities and art experiences, art mobilizes people’s senses, perceptions, memories, thoughts, and imaginations in many forms, which further stimulates people’s emotions and feelings [69]. Finally, urban public space embodies a city’s civilization and development process, and a public space that meets people’s aesthetic and living requirements can bring a good sensory experience to people. Public art that is culturally appropriate to the city has a uniquely positive impact on the city’s image, and appropriate public art can enhance the city’s image and overall competitiveness.

#### 3.3.2. The Impact of Public Art with Elements of Children’s Paintings on Public Spaces and People’s Well-Being

**Figure 7:**
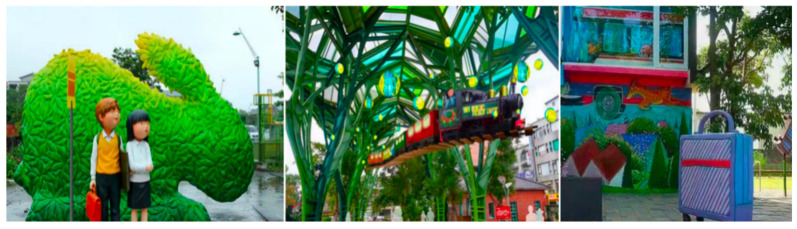
3D art at Yilan Railway Station, Yilan, Taiwan. Reprinted with permission from first author. Copyright 2018, Luo Na.

Public spaces are fundamental features of cities because they represent sites of sociability and face-to-face interaction; they provide a unique opportunity to boost the experience of SWB and M-SWB [82]. The features of children’s paintings help to enhance the inspiration of artists’ creations. Incorporating the features of children’s paintings into public artworks can enhance the artistry and innovation of public art [79]. Public art exists in people’s lives, and participation in the art can encourage a sense of personal well-being [97]. For example, the public artworks in the Yilan railway station in Taiwan (see Figure 7) are very childish and interesting, and when people walk into the station they feel that they are walking into a fairy tale world, giving them a great sense of visual stimulation and pleasure.

The design principles of interaction and innovation are essential in good public artworks. Excellent public artworks could inspire a sense of well-being [98]. The results of several studies have shown that participation in the arts has a positive impact on people’s well-being, in terms of greater satisfaction with leisure activities [99]. However, little research has been found through studies on the link between the innovativeness of public artworks and subjective well-being (SWB) [41]. It has also been noted that the redevelopment of public spaces in urban centers has left public spaces lacking in historicity [85]. This study agrees that urban green spaces (UGS) could improve the health of urban residents and contribute significantly to the well-being of people [100]. Artworks in public spaces have been suggested to add aesthetic value to their surroundings and have a positive impact on environmental quality [101]. One such special work is in the exhibition “Love will last forever” by the famous artist Yayoi Kusama. A 3-metre high pumpkin public artwork was unveiled at the Bund Financial Centre Square in Shanghai. The pumpkin holds a special place in Yayoi Kusama’s heart, resonating with the warmth of emotion and peace she feels from it. Through the newly created “Pumpkin” (2019) work, the artist hopes to infect everyone in the city with peaceful love.

Research has shown that in recent years, public artists have acquired new areas of artistic creation. For example, there is a necessary causal relationship between creativity in life and subjective well-being [41]. There are also scholars who analyze public space from different perspectives. For example, the focus is on providing convenient play spaces in densely populated cities to enhance people’s well-being [102]. In an interview by the first author, Chinese artist Qin Jizang once said, “There is no doubt that public art in public spaces can certainly enhance our sense of well-being. Childlike public artwork can create a relaxed and free space for people nowadays. It can reduce the stress in people’s lives and work, but at the moment there are too few cases of this kind in China.” Public art in public spaces is an extension of aesthetic education, it can enhance people’s perception of beauty, and childlike public art will give people a sense of pleasure, a sense of cheerfulness, and will inspire a sense of well-being.

Public art has the most basic function of artwork: the aesthetic function. Specifically, the aesthetic function includes three functions: cognition; education; and pleasure. At the same time, public art unites the cultural spirit of a city. Public art reflects a city’s cultural connotation as a significant symbol, directly reflecting the quality of life and spirituality of the city’s people. The role of public art in the city is to penetrate into the environment through its unique shape, symbolic language, artistic infection, and spatial control, so that people can gain knowledge of a certain space, stimulate their artistic emotions, and reflect the public spirit.

Public art is an important factor affecting the well-being of individuals. The subjective happiness of individuals participating in art activities will be higher than that of those not participating in art activities, and participation in public art activities will bring happiness to people. Art participation improves people’s leisure experiences. It has been studied that there are many differences in the impact of the arts on an individual’s subjective well-being in terms of satisfaction in specific areas when individuals participate in the arts. Leisure activities are a central element in influencing an individual’s subjective well-being, and the satisfaction gained from leisure experiences may be an important determinant of an individual’s happiness [50]. In everyday life, people prefer to see public art with rich connotations and that is vivid as exciting attractions to the public.

Overall, public art contributes to the improvement of urban life and the image of cities, and public artworks that are in line with modern culture can achieve cultural diffusion. As an important part of the urban landscape, urban public art is considered a furnishing in the outdoor environment, a functional and artistically symbolic work that plays a multiple role in the development of the urban environment by satisfying both the aesthetic needs of the spirit and the needs of use. Children’s paintings can bring infinite inspiration to artists, and the characteristics of children’s paintings can increase the innovative capacity of artworks. Therefore, innovative public artworks can improve the quality of public space. Moreover, public art that is childlike and highly creative can enhance the interaction with the public and promote cultural dissemination, improving residents’ sense of well-being by improving people’s leisure experience and enriching their emotional experience.

#### 3.3.3. The Process of Conceptual Framework Formation

**Figure 8:**
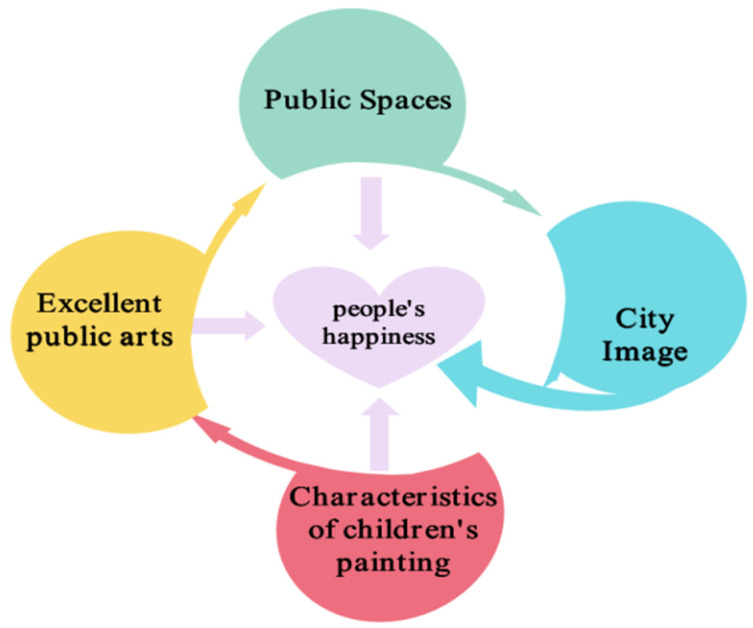
Proposed conceptual framework for public artworks in public spaces for improving people’s happiness.

In this section, the aim of this research paper is to analyze the effect of public art featuring children’s paintings on public spaces and people’s sense of well-being. The study finds that urban public spaces reflect the process of building a spiritual and material civilization in cities and that high-quality public spaces provide people with a good sensorial experience. In addition, excellent public art has a positive impact on the city’s image. Appropriate public artworks can improve the city’s image and promote comprehensive competitiveness. Childlike and creative public art can enhance interaction with the public, promote cultural dissemination, and improve residents’ sense of well-being by improving their leisure experience and enriching their emotional experience. Based on the above results, this study concludes that public artworks that feature children’s paintings and are appropriate for urban spaces can not only enhance the image of the city but also inspire a sense of well-being. Figure 8 is the subsequent proposed conceptual framework.

## 4. Discussions

Public art is based on civil society and embodies the values of democracy, openness, interaction, and sharing in contemporary public spaces, allowing for effective public participation and interaction, with accompanying institutional and procedural safeguards. However, the concept of “public art” should not be simply equated with “environmental art design”, “urban sculpture”, or “landscape art. The concept of “public art” should not be confused with “landscape art” and other concepts. Public art is not an art form but a design principle, but a design concept that is common and shared by citizens. Public art is often a kind of process art, which finds problems and tries to solve them in the dynamic passage of time, especially highlighting modern public art’s exposure and solution to social problems.

Children’s paintings occupy a unique place in the art world and can also be visually pleasing. Children’s paintings reinterpreted in public art in public spaces can create a positive and inspiring environment because they resonate with people. However, not all children’s paintings can be applied to public art nor are the features of children’s paintings appropriate for all public spaces. Public art in public spaces should conform to the principles of human ecological harmony and should respect the nature of the public environment. The public art with children’s paintings that is the focus of this study is limited to environments with a relatively relaxed atmosphere, such as parks, residential plazas, schools, and other public areas. Public spaces in office buildings and shopping plazas are also common. More serious places do not lend themselves to childlike public art.

**Figure 9:**
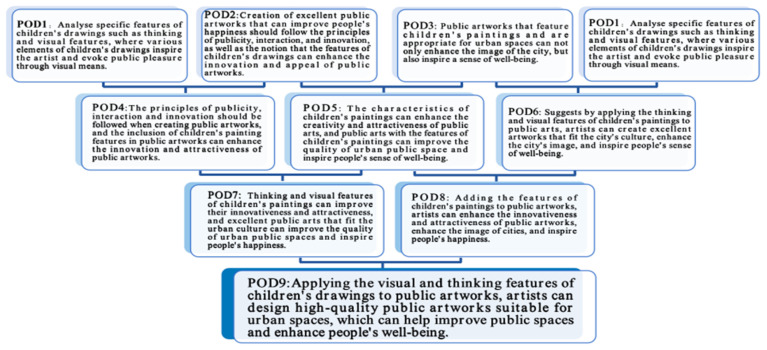
Point of Departure (POD) Tree Diagram for the impact of public artworks on public space and people’s happiness. Adapted with permission from [9]. Copyright 2018, Ibrahim and Mustafa Kamal.

This study has analyzed the impact of 2D children’s paintings on people’s well-being, examined and identified the best design principles for transforming 2D children’s paintings into 3D public artworks, and has understood the influence of public artworks featuring children’s paintings on public spaces. To address the research needs, this study analyzed and discussed each of these aspects in six separate steps. The purpose of designing the research sequence as described was to evaluate the strengths and weaknesses of various combinations of perspectives and to propose the most appropriate direction for the larger study. Referring to the POD Tree Diagram in Figure 9, this section discusses how to further synthesize the preliminary results to form a potential theoretical proposition for future research.

Through the research and discussion in Section 3.1 of this study, this study concluded that analyzing specific features of children’s paintings, such as thinking and visual features, where various elements of children’s paintings inspire the artist and evoke public pleasure through visual means (see POD1). In Section 3.2, this study concluded that the creation of excellent public artworks that can improve people’s happiness should follow the principles of publicity, interaction, and innovation, as well as the notion that features of children’s paintings can enhance the innovation and appeal of public artworks (see POD2). The synthesis of POD1 and POD2 found the principles of publicity, interaction, and innovation should be followed when creating public artworks, hence the inclusion of children’s paintings’ features in public artworks can enhance the innovation and attractiveness of public artworks (see POD4).

In Section 3.3, this study found that public artworks that feature children’s paintings and are appropriate for urban spaces can not only enhance the image of the city but also inspire a sense of well-being (see POD3). After synthesizing POD2 and POD3, this study found the characteristics of children’s paintings can enhance the creativity and attractiveness of public art, and public art with the features of children’s paintings can improve the quality of urban public space and inspire people’s sense of well-being (see POD5). After synthesizing POD3 and POD1, this study found that by applying the thinking and visual features of children’s paintings to public art, artists can create excellent artworks that fit the city’s culture, enhance the city’s image, and inspire people’s sense of well-being (see POD6).

Going through the POD Tree Diagram, after synthesizing POD4 and POD5, this study found that the thinking and visual features of children’s paintings can improve their innovativeness and attractiveness, and excellent public art that fits the urban culture can improve the quality of urban public spaces and inspire people’s happiness (see POD7). After synthesizing POD5 and POD6, this study found that by adding the features of children’s paintings to public artworks, artists can enhance the innovativeness and attractiveness of public artworks, enhance the image of cities, and inspire people’s happiness (see POD8).

**Figure 10:**
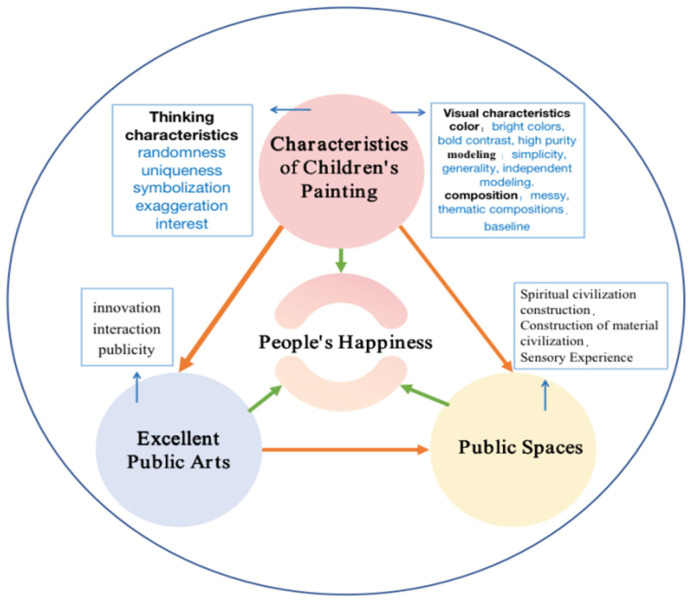
Proposed conceptual framework for public art in public spaces for improving people’s happiness.

As the conclusion of this synthesis exercise between POD7 and POD8, this study found that by applying the visual and thinking features of children’s paintings to public artworks, artists can design high-quality public artworks suitable for urban spaces, which can help improve public spaces and enhance people’s well-being (see POD9). Based on POD9, this study posits that a potential solution can be achieved by creating an integrated process for improving public spaces by transforming 2D children’s paintings into 3D public artworks to enhance people’s well-being. Figure 10 shows the subsequent proposed conceptual framework.

## 5. Conclusions

The aim of this study is to improve public spaces for elevating people’s happiness by transforming 3D public artworks from 2D children’s paintings. The study included elements of 2D children’s paintings, transformation into 3D public art, and improvement in public spaces. Regarding the content of the 2D children’s paintings, the study found that using the thinking and visual features of children’s paintings in artwork, can stimulate people’s happiness by visual means and bring inspiration to artists. With regard to discussing the transformation into 3D public artwork, the study found that analyzing specific features of children’s paintings, such as the thinking and visual features, meant that various elements of children’s paintings could inspire the artist and evoke public pleasure through visual means. Moreover, in discussing the improvement of public spaces, the study found that public artworks that feature children’s paintings and are appropriate for urban spaces can not only enhance the image of the city but also inspire a sense of well-being. Further synthesis of the above results concludes the conceptual framework for public artworks in public spaces for improving people’s happiness. The proposed conceptual framework recommends that by applying the visual and thinking features of children’s paintings to public art, artists can design high-quality artworks suitable for city space, which helps to improve the public spaces for people’s happiness. The research results are significant because they help artists to create more high-quality public artworks for urban public spaces in order to evoke people’s happiness. This study recommends further research into how public art can promote public spaces and shape the urban culture. This study contributes in enhancing the quality of public art and public spaces, and inspiring a sense of well-being among citizens through the use of appropriate public art.

## Data Availability

The data presented in this study are available on request from the corresponding author. The data are not publicly available due to the restriction on copyright.
